# Supplementary material for: Modeling glioblastoma heterogeneity as a dynamic network of cell states
Source: Mol Syst Biol. 2021 Sep 16;17(9):e10105. doi: 10.15252/msb.202010105 (PMC8444284; doi:10.15252/msb.202010105)
Supplement: Supplementary file 5 — Source Data for Figure 3 [file MSB-17-e10105-s001.zip › Figure3A_sourcedata/GSEA_3065/hallmarks_state2.GseaPreranked.1623416269049/HALLMARK_MITOTIC_SPINDLE.html]

Details for gene set HALLMARK\_MITOTIC\_SPINDLE[GSEA]

|  || Dataset | state2 |
| Phenotype | NoPhenotypeAvailable |
| Upregulated in class | na\_pos |
| GeneSet | HALLMARK\_MITOTIC\_SPINDLE |
| Enrichment Score (ES) | 0.5332562 |
| Normalized Enrichment Score (NES) | 1.9045346 |
| Nominal p-value | 0.0 |
| FDR q-value | 0.0 |
| FWER p-Value | 0.0 |
Table: GSEA Results Summary

  

Fig 1: Enrichment plot: HALLMARK\_MITOTIC\_SPINDLE      
 Profile of the Running ES Score & Positions of GeneSet Members on the Rank Ordered List

  

| PROBE | GENE SYMBOL | GENE\_TITLE | RANK IN GENE LIST | RANK METRIC SCORE | RUNNING ES | CORE ENRICHMENT || 1 | TOP2A |  |  | 15 | 0.596 | 0.0265 | Yes |
| 2 | CDK1 |  |  | 18 | 0.577 | 0.0534 | Yes |
| 3 | TPX2 |  |  | 29 | 0.531 | 0.0774 | Yes |
| 4 | CENPF |  |  | 34 | 0.518 | 0.1014 | Yes |
| 5 | SMC4 |  |  | 42 | 0.489 | 0.1237 | Yes |
| 6 | NUSAP1 |  |  | 47 | 0.483 | 0.1460 | Yes |
| 7 | BIRC5 |  |  | 61 | 0.443 | 0.1655 | Yes |
| 8 | FBXO5 |  |  | 67 | 0.420 | 0.1847 | Yes |
| 9 | LMNB1 |  |  | 69 | 0.417 | 0.2043 | Yes |
| 10 | KIF4A |  |  | 84 | 0.393 | 0.2213 | Yes |
| 11 | KIF15 |  |  | 86 | 0.392 | 0.2397 | Yes |
| 12 | CENPE |  |  | 105 | 0.372 | 0.2552 | Yes |
| 13 | ANLN |  |  | 111 | 0.358 | 0.2716 | Yes |
| 14 | NDC80 |  |  | 115 | 0.354 | 0.2880 | Yes |
| 15 | KIF23 |  |  | 117 | 0.351 | 0.3044 | Yes |
| 16 | KIF11 |  |  | 129 | 0.335 | 0.3190 | Yes |
| 17 | KIF22 |  |  | 154 | 0.306 | 0.3308 | Yes |
| 18 | ECT2 |  |  | 171 | 0.297 | 0.3431 | Yes |
| 19 | CCNB2 |  |  | 190 | 0.286 | 0.3547 | Yes |
| 20 | SMC3 |  |  | 197 | 0.283 | 0.3673 | Yes |
| 21 | KIF20B |  |  | 208 | 0.278 | 0.3794 | Yes |
| 22 | SMC1A |  |  | 249 | 0.253 | 0.3870 | Yes |
| 23 | KIF2C |  |  | 252 | 0.251 | 0.3986 | Yes |
| 24 | SAC3D1 |  |  | 263 | 0.246 | 0.4091 | Yes |
| 25 | RACGAP1 |  |  | 333 | 0.222 | 0.4122 | Yes |
| 26 | BUB1 |  |  | 335 | 0.222 | 0.4225 | Yes |
| 27 | PLK1 |  |  | 364 | 0.213 | 0.4295 | Yes |
| 28 | DLGAP5 |  |  | 371 | 0.211 | 0.4388 | Yes |
| 29 | AURKA |  |  | 375 | 0.210 | 0.4484 | Yes |
| 30 | CDK5RAP2 |  |  | 423 | 0.195 | 0.4525 | Yes |
| 31 | BRCA2 |  |  | 427 | 0.193 | 0.4613 | Yes |
| 32 | YWHAE |  |  | 511 | 0.178 | 0.4607 | Yes |
| 33 | PRC1 |  |  | 514 | 0.178 | 0.4689 | Yes |
| 34 | CEP57 |  |  | 531 | 0.176 | 0.4755 | Yes |
| 35 | ARHGDIA |  |  | 546 | 0.173 | 0.4821 | Yes |
| 36 | MAPRE1 |  |  | 569 | 0.168 | 0.4877 | Yes |
| 37 | CKAP5 |  |  | 596 | 0.164 | 0.4926 | Yes |
| 38 | KIF5B |  |  | 604 | 0.163 | 0.4995 | Yes |
| 39 | MARCKS |  |  | 630 | 0.159 | 0.5043 | Yes |
| 40 | RFC1 |  |  | 655 | 0.156 | 0.5090 | Yes |
| 41 | STAU1 |  |  | 659 | 0.155 | 0.5160 | Yes |
| 42 | NEK2 |  |  | 805 | 0.138 | 0.5068 | Yes |
| 43 | SASS6 |  |  | 811 | 0.137 | 0.5128 | Yes |
| 44 | TTK |  |  | 831 | 0.135 | 0.5171 | Yes |
| 45 | PCNT |  |  | 844 | 0.133 | 0.5220 | Yes |
| 46 | PCM1 |  |  | 861 | 0.131 | 0.5265 | Yes |
| 47 | PREX1 |  |  | 890 | 0.129 | 0.5296 | Yes |
| 48 | UXT |  |  | 912 | 0.126 | 0.5333 | Yes |
| 49 | CENPJ |  |  | 1192 | 0.105 | 0.5081 | No |
| 50 | EPB41L2 |  |  | 1200 | 0.105 | 0.5122 | No |
| 51 | WASL |  |  | 1207 | 0.104 | 0.5165 | No |
| 52 | PPP4R2 |  |  | 1228 | 0.103 | 0.5192 | No |
| 53 | RALBP1 |  |  | 1253 | 0.101 | 0.5214 | No |
| 54 | CDC27 |  |  | 1319 | 0.097 | 0.5189 | No |
| 55 | FARP1 |  |  | 1441 | 0.090 | 0.5101 | No |
| 56 | CEP192 |  |  | 1597 | 0.082 | 0.4972 | No |
| 57 | ARF6 |  |  | 1611 | 0.081 | 0.4996 | No |
| 58 | CEP72 |  |  | 1625 | 0.081 | 0.5020 | No |
| 59 | KATNA1 |  |  | 1711 | 0.076 | 0.4964 | No |
| 60 | ROCK1 |  |  | 1729 | 0.076 | 0.4982 | No |
| 61 | CDC42 |  |  | 1773 | 0.074 | 0.4970 | No |
| 62 | INCENP |  |  | 1833 | 0.071 | 0.4940 | No |
| 63 | CNTRL |  |  | 1880 | 0.069 | 0.4922 | No |
| 64 | TBCD |  |  | 2125 | 0.060 | 0.4687 | No |
| 65 | ARHGAP10 |  |  | 2253 | 0.055 | 0.4576 | No |
| 66 | DYNLL2 |  |  | 2283 | 0.054 | 0.4570 | No |
| 67 | CAPZB |  |  | 2286 | 0.054 | 0.4594 | No |
| 68 | NET1 |  |  | 2298 | 0.054 | 0.4607 | No |
| 69 | TUBGCP3 |  |  | 2300 | 0.054 | 0.4632 | No |
| 70 | MID1IP1 |  |  | 2313 | 0.053 | 0.4644 | No |
| 71 | WASF2 |  |  | 2359 | 0.052 | 0.4619 | No |
| 72 | KNTC1 |  |  | 2440 | 0.050 | 0.4556 | No |
| 73 | BIN1 |  |  | 2492 | 0.048 | 0.4524 | No |
| 74 | GEMIN4 |  |  | 2615 | 0.044 | 0.4413 | No |
| 75 | KATNB1 |  |  | 2741 | 0.040 | 0.4297 | No |
| 76 | CEP131 |  |  | 2773 | 0.040 | 0.4282 | No |
| 77 | EPB41 |  |  | 2818 | 0.038 | 0.4253 | No |
| 78 | ESPL1 |  |  | 2838 | 0.038 | 0.4250 | No |
| 79 | WASF1 |  |  | 2850 | 0.038 | 0.4256 | No |
| 80 | ALMS1 |  |  | 2919 | 0.036 | 0.4199 | No |
| 81 | FSCN1 |  |  | 2952 | 0.035 | 0.4181 | No |
| 82 | CDC42EP1 |  |  | 3034 | 0.033 | 0.4110 | No |
| 83 | EZR |  |  | 3039 | 0.033 | 0.4121 | No |
| 84 | NIN |  |  | 3079 | 0.032 | 0.4094 | No |
| 85 | SOS1 |  |  | 3120 | 0.031 | 0.4065 | No |
| 86 | MYH10 |  |  | 3132 | 0.031 | 0.4068 | No |
| 87 | ARHGAP5 |  |  | 3234 | 0.029 | 0.3973 | No |
| 88 | RANBP9 |  |  | 3241 | 0.029 | 0.3980 | No |
| 89 | CNTROB |  |  | 3328 | 0.027 | 0.3900 | No |
| 90 | NCK2 |  |  | 3539 | 0.023 | 0.3684 | No |
| 91 | TUBGCP5 |  |  | 3623 | 0.022 | 0.3604 | No |
| 92 | TLK1 |  |  | 3656 | 0.021 | 0.3580 | No |
| 93 | TUBD1 |  |  | 3707 | 0.020 | 0.3535 | No |
| 94 | HOOK3 |  |  | 3838 | 0.018 | 0.3403 | No |
| 95 | MAP1S |  |  | 3984 | 0.016 | 0.3254 | No |
| 96 | ARHGEF12 |  |  | 4018 | 0.015 | 0.3226 | No |
| 97 | CCDC88A |  |  | 4035 | 0.015 | 0.3216 | No |
| 98 | PAFAH1B1 |  |  | 4126 | 0.014 | 0.3125 | No |
| 99 | RASA2 |  |  | 4350 | 0.010 | 0.2888 | No |
| 100 | LRPPRC |  |  | 4429 | 0.008 | 0.2808 | No |
| 101 | CD2AP |  |  | 4604 | 0.006 | 0.2623 | No |
| 102 | ATG4B |  |  | 4715 | 0.004 | 0.2506 | No |
| 103 | CYTH2 |  |  | 4839 | 0.002 | 0.2374 | No |
| 104 | TUBGCP2 |  |  | 4895 | 0.001 | 0.2315 | No |
| 105 | ARL8A |  |  | 5135 | -0.002 | 0.2058 | No |
| 106 | RAPGEF6 |  |  | 5326 | -0.005 | 0.1855 | No |
| 107 | CDC42EP4 |  |  | 5335 | -0.005 | 0.1849 | No |
| 108 | PLEKHG2 |  |  | 5383 | -0.006 | 0.1801 | No |
| 109 | BCL2L11 |  |  | 5510 | -0.009 | 0.1669 | No |
| 110 | MYO9B |  |  | 5595 | -0.010 | 0.1583 | No |
| 111 | PALLD |  |  | 5615 | -0.010 | 0.1568 | No |
| 112 | CSNK1D |  |  | 5691 | -0.011 | 0.1492 | No |
| 113 | PCGF5 |  |  | 5711 | -0.012 | 0.1477 | No |
| 114 | HDAC6 |  |  | 5777 | -0.013 | 0.1413 | No |
| 115 | MAP3K11 |  |  | 5782 | -0.013 | 0.1414 | No |
| 116 | PIF1 |  |  | 5869 | -0.014 | 0.1328 | No |
| 117 | STK38L |  |  | 5935 | -0.015 | 0.1265 | No |
| 118 | ABL1 |  |  | 5952 | -0.016 | 0.1255 | No |
| 119 | LATS1 |  |  | 5984 | -0.016 | 0.1230 | No |
| 120 | SSH2 |  |  | 6043 | -0.017 | 0.1175 | No |
| 121 | ABR |  |  | 6092 | -0.018 | 0.1132 | No |
| 122 | TAOK2 |  |  | 6113 | -0.018 | 0.1119 | No |
| 123 | ACTN4 |  |  | 6190 | -0.020 | 0.1046 | No |
| 124 | CTTN |  |  | 6421 | -0.024 | 0.0809 | No |
| 125 | RAB3GAP1 |  |  | 6489 | -0.025 | 0.0748 | No |
| 126 | KIF3B |  |  | 6503 | -0.025 | 0.0746 | No |
| 127 | PXN |  |  | 6551 | -0.026 | 0.0708 | No |
| 128 | SHROOM2 |  |  | 6628 | -0.028 | 0.0639 | No |
| 129 | CLIP2 |  |  | 6639 | -0.028 | 0.0641 | No |
| 130 | TUBGCP6 |  |  | 6711 | -0.030 | 0.0579 | No |
| 131 | RASA1 |  |  | 6745 | -0.030 | 0.0557 | No |
| 132 | ABI1 |  |  | 6766 | -0.031 | 0.0550 | No |
| 133 | ALS2 |  |  | 6831 | -0.032 | 0.0496 | No |
| 134 | ARHGEF3 |  |  | 6923 | -0.035 | 0.0414 | No |
| 135 | TIAM1 |  |  | 6948 | -0.035 | 0.0405 | No |
| 136 | FGD4 |  |  | 6960 | -0.035 | 0.0410 | No |
| 137 | BCR |  |  | 7063 | -0.038 | 0.0317 | No |
| 138 | NEDD9 |  |  | 7098 | -0.039 | 0.0299 | No |
| 139 | KIFAP3 |  |  | 7156 | -0.041 | 0.0257 | No |
| 140 | KIF1B |  |  | 7160 | -0.041 | 0.0273 | No |
| 141 | MARK4 |  |  | 7168 | -0.041 | 0.0285 | No |
| 142 | CLASP1 |  |  | 7174 | -0.041 | 0.0299 | No |
| 143 | CDC42BPA |  |  | 7216 | -0.042 | 0.0274 | No |
| 144 | ARHGEF11 |  |  | 7361 | -0.046 | 0.0141 | No |
| 145 | ARFIP2 |  |  | 7395 | -0.047 | 0.0127 | No |
| 146 | KIF3C |  |  | 7422 | -0.048 | 0.0122 | No |
| 147 | RABGAP1 |  |  | 7535 | -0.051 | 0.0025 | No |
| 148 | NCK1 |  |  | 7576 | -0.053 | 0.0006 | No |
| 149 | NUMA1 |  |  | 7579 | -0.053 | 0.0029 | No |
| 150 | ARHGEF7 |  |  | 7591 | -0.053 | 0.0042 | No |
| 151 | BCAR1 |  |  | 7653 | -0.055 | 0.0002 | No |
| 152 | PDLIM5 |  |  | 7663 | -0.055 | 0.0018 | No |
| 153 | RICTOR |  |  | 7746 | -0.058 | -0.0043 | No |
| 154 | KLC1 |  |  | 7942 | -0.064 | -0.0223 | No |
| 155 | CLIP1 |  |  | 7967 | -0.066 | -0.0218 | No |
| 156 | PKD2 |  |  | 7992 | -0.067 | -0.0212 | No |
| 157 | RASAL2 |  |  | 8072 | -0.070 | -0.0265 | No |
| 158 | ARHGEF2 |  |  | 8108 | -0.072 | -0.0269 | No |
| 159 | TSC1 |  |  | 8114 | -0.072 | -0.0240 | No |
| 160 | TRIO |  |  | 8122 | -0.073 | -0.0214 | No |
| 161 | RHOT2 |  |  | 8124 | -0.073 | -0.0180 | No |
| 162 | SUN2 |  |  | 8189 | -0.077 | -0.0213 | No |
| 163 | ARFGEF1 |  |  | 8352 | -0.086 | -0.0348 | No |
| 164 | NOTCH2 |  |  | 8358 | -0.086 | -0.0313 | No |
| 165 | ITSN1 |  |  | 8414 | -0.090 | -0.0330 | No |
| 166 | FLNB |  |  | 8416 | -0.090 | -0.0289 | No |
| 167 | CEP250 |  |  | 8431 | -0.091 | -0.0261 | No |
| 168 | ARHGAP29 |  |  | 8476 | -0.094 | -0.0264 | No |
| 169 | APC |  |  | 8542 | -0.100 | -0.0287 | No |
| 170 | NF1 |  |  | 8661 | -0.108 | -0.0364 | No |
| 171 | MYH9 |  |  | 8778 | -0.119 | -0.0433 | No |
| 172 | FLNA |  |  | 8824 | -0.126 | -0.0423 | No |
| 173 | DLG1 |  |  | 8857 | -0.130 | -0.0396 | No |
| 174 | OPHN1 |  |  | 8872 | -0.133 | -0.0348 | No |
| 175 | MID1 |  |  | 8907 | -0.137 | -0.0321 | No |
| 176 | SPTAN1 |  |  | 8923 | -0.138 | -0.0272 | No |
| 177 | SORBS2 |  |  | 8978 | -0.145 | -0.0262 | No |
| 178 | AKAP13 |  |  | 8995 | -0.148 | -0.0209 | No |
| 179 | SPTBN1 |  |  | 9111 | -0.169 | -0.0254 | No |
| 180 | DYNC1H1 |  |  | 9231 | -0.205 | -0.0286 | No |
| 181 | GSN |  |  | 9238 | -0.206 | -0.0196 | No |
| 182 | VCL |  |  | 9405 | -0.389 | -0.0192 | No |
| 183 | DST |  |  | 9422 | -0.485 | 0.0019 | No |
Table: GSEA details [plain text format]

  

Fig 2: HALLMARK\_MITOTIC\_SPINDLE: Random ES distribution      
 Gene set null distribution of ES for **HALLMARK\_MITOTIC\_SPINDLE**
